# Induction of Protective Immunity against Toxoplasmosis in BALB/c Mice Vaccinated with *Toxoplasma gondii* Rhoptry-1

**DOI:** 10.3389/fmicb.2016.00808

**Published:** 2016-05-27

**Authors:** Parthasarathy Sonaimuthu, Xiao T. Ching, Mun Y. Fong, Ramaswamy Kalyanasundaram, Yee L. Lau

**Affiliations:** ^1^Department of Parasitology, Faculty of Medicine, University of MalayaKuala Lumpur, Malaysia; ^2^Department of Biomedical Sciences, College of Medicine, University of Illinois at Chicago, ChicagoIL, USA

**Keywords:** *Toxoplasma gondii*, vaccine, ROP1, DNA plasmid, recombinant protein

## Abstract

*Toxoplasma gondii* is the causative agent for toxoplasmosis. The rhoptry protein 1 (ROP1) is secreted by rhoptry, an apical secretory organelle of the parasite. ROP1 plays an important role in host cell invasion. In this study, the efficacy of ROP1 as a vaccine candidate against toxoplasmosis was evaluated through intramuscular or subcutaneous injection of BALB/c mice followed by immunological characterization (humoral- and cellular-mediated) and lethal challenge against virulent *T. gondii* RH strain in BALB/c mice. Briefly, a recombinant DNA plasmid (pVAX1-GFP-ROP1) was expressed in CHO cells while expression of recombinant ROP1 protein (rROP1) was carried out in *Escherichia coli* expression system. Immunization study involved injection of the recombinant pVAX1-ROP1 and purified rROP1 into different group of mice. Empty vector and PBS served as two different types of negative controls. Results obtained demonstrated that ROP1 is an immunogenic antigen that induced humoral immune response whereby detection of a protein band with expected size of 43 kDa was observed against vaccinated mice sera through western blot analysis. ROP1 antigen was shown to elicit cellular-mediated immunity as well whereby stimulated splenocytes with total lysate antigen (TLA) and rROP1 from pVAX1-ROP1 and rROP1-immunized mice, respectively, readily proliferated and secreted large amount of IFN-γ (712 ± 28.1 pg/ml and 1457 ± 31.19 pg/ml, respectively) and relatively low IL-4 level (94 ± 14.5 pg/ml and 186 ± 14.17 pg/ml, respectively). These phenomena suggested that Th1-favored immunity was being induced. Vaccination with ROP1 antigen was able to provide partial protection in the vaccinated mice against lethal challenge with virulent RH strain of tachyzoites. These findings proposed that the ROP1 antigen is a potential candidate for the development of vaccine against toxoplasmosis.

## Introduction

*Toxoplasma gondii* is an obligate intracellular parasite that infects various cell types to cause an infection known as toxoplasmosis (Kim and Weiss, 2004). Although the infection is usually asymptomatic, it may sometimes lead to severe complications, including brain lesions, encephalitis, and neurological diseases. The risk is highest in immunocompromised patients. Infants affected by congenital toxoplasmosis can develop hydrocephalus, convulsions, microcephaly, motor retardation, anemia, and intracerebral calcification. *T. gondii* infections in livestock can result in abortion or stillbirth, causing major economic losses worldwide (Buxton, 1998).

Current therapies against *T. gondii* are toxic and expensive. They are designed to control newly acquired infections, but not for treating chronic toxoplasmosis. Thus, vaccination is considered as an effective approach for preventing *T. gondii* infection. However, the only available vaccine for toxoplasmosis to date is derived from live attenuated *T. gondii* (non-cyst-forming S48 strain). It is only used for sheep in Europe and New Zealand (Buxton et al., 1991; Jongert et al., 2009). It is not suitable for human use because of the risks associated with reversion of the parasite to its pathogenic form (Weeks-Levy et al., 1991; Bourguin et al., 1993). To date, various *T. gondii* antigens, such as microneme proteins (Lourenco et al., 2006; Peng et al., 2009; Yan et al., 2012), dense granule proteins (Golkar et al., 2007; Jongert et al., 2008; Hiszczynska-Sawicka et al., 2011b; Sun et al., 2011), rhoptry antigens (Chen et al., 2003; Yuan et al., 2011a; Dziadek et al., 2012), matrix proteins (Di Cristina et al., 2004), and surface antigens (Khan et al., 1991; Mevelec et al., 2005; Lau et al., 2011) have been assessed as potential vaccination candidates.

DNA vaccines are considered as an alternative approach to live, attenuated vaccines because they can elicit long-lived immune responses in animal (Robinson, 1999; Gurunathan et al., 2000). Moreover, these vaccines have been seen to be safe and effective in controlling *T. gondii* infection (Liu et al., 2012). Cellular immune responses generated through immunization are particularly important for combating *T. gondii*, and intramuscular DNA vaccines are known to induce both cellular and humoral immunity (Denkers and Gazzinelli, 1998). Subunit vaccines are known to trigger predominantly humoral immune responses and this is characterized by isotopic diversity of the antibodies with different effector functions; in particular complement activation and binding to phagocytic and killer cells through Fc receptors (Janeway et al., 2001). Efficient humoral responses require cooperation between B and specific T helper (Th) cells. The cells are divided into two distinct subpopulations, based on the cytokines they secrete and their effector functions. For example, Th1 cells produce mainly IL-2, IFN-γ, and TNF-β, and they stimulate a cellular delayed type hypersensitivity response. In contrast, Th2 cells produce IL-4, IL-5, IL-10, and IL-13 cytokines (Romagnani, 1994; Abbas et al., 1996).

Host cell invasion by *T. gondii* is mediated by proteins which are present in the apical complex secretory organelles (micronemes, dense granules, and rhoptries; Saeij et al., 2006). The rhoptries are involved in the formation of the parasitophorous vacuoles in which the parasites proliferate, thus avoiding host immune defenses (Carey et al., 2004; Dlugonska, 2008). Numerous investigations have been carried out on rhoptry protein 1 (ROP1), ROP2, ROP16, and ROP18 as potential vaccine candidates (Chen et al., 2001; Leyva et al., 2001; Yuan et al., 2011a,b).

DNA vaccine of ROP1 has been shown to elicit immunity in animal models (Chen et al., 2001; Guo et al., 2001). ROP1 in combination with SAG1 has been shown to induce protective immunity in mice (Chen et al., 2003). DNA vaccine combining *ROP1* gene with ovine CD154 triggers a mixed Th1/Th2 immune response in sheep, whereas ROP1 alone induces only Th1-specific immunity (Hiszczynska-Sawicka et al., 2011a). A DNA vaccine cocktail containing recombinant ROP1 (rROP1) and GRA7 was also tested with and without adjuvant pIL-12 in mice. The multivalent vaccine ROP1-GRA7 with pIL12 showed stronger Th1 immune response and protective efficiency than the ROP1 alone (Quan et al., 2012). Studies thus far only report the type of immune responses triggered by ROP1-based DNA vaccine, and no challenge experiments against acute infection have been conducted. The present study is the first to demonstrate the immuno-protectivity of ROP1-based DNA vaccine and rROP1 against acute *Toxoplasma* infection in BALB/c mice.

## Materials and Methods

### Mice

Female BALB/c mice (5–6 weeks old) were purchased from University of Malaya Animal Experimental Center and housed in a pathogen-free environment. Experiments involving mice model were performed in compliance with the animal ethics approved by Institutional Animal Care and Use Committee (IACUC) of University of Malaya, Faculty of Medicine (2014-06-03/PARA/R/CXT).

### Parasites

Highly virulent *T. gondii* tachyzoites (RH strain) were maintained via successive intraperitoneal passage in mice. Parasites were collected from the intraperitoneal fluid, washed with phosphate buffered saline (PBS), and filtered through 3 μm polycarbonate filters. The filtrate obtained was centrifuged at 1,500 rpm for 10 min and re-suspended in PBS prior to use.

### Preparation of Total Lysate Antigen (TLA)

Total lysate antigen (TLA) of the parasite was extracted from the suspension of tachyzoites through repeated freeze-thaw methods (5–10 cycles of freezing in liquid nitrogen and thawing in 37°C water bath with 2–3 min each step) followed by centrifugation of the lysate at 3000 × *g* for 15 min at 4°C. Supernatant containing TLA was collected and stored at -80°C until used.

### Construction of Recombinant Plasmids

#### Eukaryotic Expression Plasmids

The 1,191 bp *ROP1* coding sequence (GenBank ID: M71274) was amplified by PCR from genomic DNA of *T. gondii* tachyzoites using gene-specific forward (5′-CGCGGATCCACGATGGAGCAAAGGCTGCCAA-3′) and reverse (5′-CGGAATTCTTATTGCGATCCATCATCCTGC-3′) primers, introducing *Bam* HI and *Eco* R1 restriction sites (as underlined). The PCR product was cloned into the respective restriction sites of pVAX1 (Invitrogen, USA) and pVAX1-GFP vectors. The constructs were verified by sequencing in both directions to ensure fidelity (GenScript Inc., Piscataway, NJ, USA).

#### Prokaryotic Expression Plasmids

The 1,191 bp *ROP1* coding sequence (GenBank ID: M71274) was amplified by PCR from genomic DNA of the *T. gondii* tachyzoites with gene-specific forward (5′-GAATTCCTACTTGTTCTCTCTGTG-3′) and reverse (5′-GGATCCTTGCGATCCATCATCCTG-3′) primers, introducing *Eco* R1 and *Bam* HI restriction sites (as underlined). The PCR product was cloned into the respective restriction sites of pCold I vector (Invitrogen, USA). The constructs were verified by sequencing in both directions to ensure fidelity (Bioneer Corporation, South Korea). Both the rROP1 construct and non-recombinant pCold I plasmid were transformed into the prokaryotic expression host, *E. coli* BL21 (DE3) prior to protein expression.

### *In Vitro* Expression of pVAX1-GFP-ROP1

Chinese Hamster Ovary (CHO) cells were transfected with pVAX1-GFP-ROP1 plasmid (3 μg/μl) using TurboFect (Fermentas Inc., Glen Burnie, MA, USA) according to the manufacturer’s instructions. Expression of green fluorescent protein (GFP)-tagged ROP1 was observed under inverted florescence microscope (Olympus, Hicksville, NY) at 24, 48, and 72 h post transfection. The expression was also detected by western blot assay using monoclonal anti-GFP antibody (Invitrogen, Grand Island, NY, USA) and toxoplasmosis-positive patient serum (Diagnostic Laboratory [Para: SEAD], Department of Parasitology, University of Malaya). CHO cells transfected with pVAX1-GFP vector was included as positive control.

### Recombinant Protein Expression of pCold I-ROP1 (rROP1) in BL21 (DE3)

A single colony of pCold I-ROP1 was inoculated into 5 ml of LB broth supplemented with ampicillin (100 μg/ml) and chloramphenicol (34 μg/ml) before growing overnight at 37°C, with 250 rpm shaking. One percent of the overnight culture was inoculated into 10 ml of LB medium and grown at 37°C, 250 rpm until the OD600 reached 0.5–0.6. The culture was then incubated at 15°C for 30 min followed by the addition of 1 mM isopropyl-b-D-thiogalactopyranoside (IPTG). The cells were again allowed to grow overnight with constant shaking at 15°C, 250 rpm. The culture was harvested by centrifugation at 10,000 rpm for 10 min the next day. Cell pellet obtained was subjected to purification.

### One-Step Protein Purification of rROP1

Protein expressed was purified under native condition using ProBond^TM^ Purification system (Invitrogen, USA) according to the manufacturer’s instructions. The cell pellet obtained was re-suspended in the binding buffer before subjected to sonication for 60 s at 10 s interval. The sonicated cell lysate was then centrifuged at 10,000 × *g* for 30 min, 4°C. Supernatant was collected and loaded into the pre-equilibrated purification column containing 2 ml nickel-nitrilotriacetic acid (Ni-NTA) resin (Invitrogen, USA). Supernatant containing rROP1 was allowed to bind to the resin at room temperature for 3 h. After binding, the resin with bound protein was washed with buffers containing imidazole with increasing concentrations (5, 10, and 15 mM, respectively) in order to remove contaminant proteins that bound unspecifically to the resin. Target protein was eventually eluted with elution buffer containing 200 mM imidazole. Eluted rROP1 was subjected to dialysis overnight with PBS to remove unwanted salt from the protein. Purity and identity of the purified protein was analyzed through SDS-PAGE and western blot analysis.

### SDS-PAGE and Western Blot Analysis

Purified rROP1 was resolved by SDS-PAGE on 12% polyacrylamide gel and transferred onto methanol-activated polyvinylidene difluoride (PVDF; Bio-Rad, USA) membrane. The membrane were incubated with blocking solution (5% non-fat skim milk in Tris Buffered Saline, TBS) for 2 h at room temperature with constant shaking and were subsequently probed with monoclonal anti-His (Invitrogen, USA; 1:5000) for 2 h. The membrane was washed and then incubated for 1 h with biotinylated goat anti-mouse IgG (KPL, USA; 1:2500) secondary antibody. Lastly, the membrane was washed and incubated with streptavidin-alkaline phosphatase (KPL, USA; 1:2,500) at room temperature for 1 h followed by detection using 5-bromo-4-chloro-3-indolyphosphate/nitro blue tetrazolium (BCIP/NBT; Sigma, USA).

### Immunization of Mice

Six groups of BALB/c mice (*n* = 15 each) were vaccinated three times at 2 weeks intervals; (1) pVAX1-ROP1 (100 μg), (2) pVAX1 (100 μg), (3) PBS, (4) rROP1 (10 μg), (5) pCold I, and (6) PBS. Group 1–3 involved intramuscular injection while group 4–6 were injected subcutaneously. Injection samples for group 4–6 were mixed evenly with the adjuvants (complete freund’s, CFA for prime injection; incomplete freund’s, IFA for booster injections) before immunizing the mice. Mice sera from each group were collected via tail-bleeding at the end of each interval. The sera were examined for reaction against extracted TLA or purified rROP1 via western blot assay. Two weeks after final injection, three mice from each group were sacrificed by cervical dislocation and the spleens were collected aseptically for splenocytes isolation.

### *In Vitro* Lymphocyte Proliferation Assay

Briefly, splenocytes suspensions were prepared from three immunized mice in each group by pushing the spleens through a wire mesh. After the red blood cells (RBCs) were removed using standard Ammonium–Chloride–Potassium (ACK) lysis buffer, the splenocytes were suspended in RPMI 1640 medium supplemented with 10% fetal calf serum (FCS), 0.5% gentamicin, and 1% glutamine. The cells were then plated in 96-well Costar plates at a density of 2 × 10^5^ cells per well and cultured with 10 μg/ml TLA or rROP1, 5 μg/ml concanavalin A (Con A), or with medium alone (negative control) at 37°C with 5% CO_2_. Splenocytes proliferation was measured using bromodeoxyuridine (BrdU) colorimetric assay according to the manufacturer’s instructions (Roche, Indianapolis, IN, USA). Stimulation index (SI) was calculated as follow.

Stimulation index (SI)=mean OD570 values of stimulated cellsmean OD570 values of unstimulated cells

All assays were performed in triplicate.

### Cytokine Assays

Supernatants were harvested from the splenocytes cultures 72 h after stimulation with TLA or rROP1, ConA, or medium alone. Cytokines were measured using a commercial enzyme-linked immunosorbent assay (ELISA) kit (BD Biosciences, San Jose, CA, USA). The concentration of interleukin-4 (IL-4) and gamma interferon (IFN-γ) in supernatants were determined using standard reference curves constructed with known amounts of recombinant mouse IFN-γ and IL-4. Sensitivities of the assays are <5 pg/ml and <10 pg/ml for IL-4 and IFN-γ respectively.

### Challenge with *T. gondii*

The remaining 12 immunized mice from each group were challenged with 1 × 10^3^ live tachyzoites by intraperitoneal injections 2 weeks after final injection. The challenged mice were being monitored over a period of 28 days and their survival rates were calculated. After 28 days all the surviving mice were humanely killed by CO_2_ asphyxiation.

### Statistical Analysis

Statistical significance of the data obtained was tested using Students *t*-test or ANOVA with SPSS software. P values of <0.05 were considered significant. The graph for the survival curve was drawn based on the Kaplan–Meier approach (Kaplan and Meier, 1958). Survival rates were statistically compared using the chi-square (χ^2^) test.

## Results

### Construction of Recombinant Plasmids

Full length open reading frame of *ROP1* gene was successfully amplified from *T. gondii* genomic DNA producing an amplified product of 1191 bp (data not shown). The PCR fragment was cloned into eukaryotic expression vector (pVAX1 and pVAX1-GFP) and prokaryotic expression vector (pCold I) before transforming into TOP10F’ and BL21 (DE3) bacterial cells, respectively. The nucleotide sequence of the recombinant clones (pVAX1-ROP1, pVAX1-GFP-ROP, and pCold I-ROP1) obtained were analyzed with BLAST alignment tool which revealed that they shared 100% similarity of the published coding sequence of ROP1 (data not shown).

### Eukaryotic Expression of pVAX1-GFP-ROP1 in CHO Cells

*ROP1* gene was expressed in CHO cells before performing DNA immunization study in order to prove that the constructed pVAX1-ROP1 could be expressed constitutively and that the expression would not affect the viability of the cells. Transfection of pVAX1-ROP1 into CHO cell was carried out using Lipofectamine, a positively charged liposome that interacted with the negatively charged pVAX1-ROP1 which then facilitated its entry into the cell by overcoming the electrostatic repulsion. Entry of the transfection complex into CHO cell occurred through endocytosis, a process of DNA uptake by the cell membrane followed by diffusion through cytoplasm to reach the cell nucleus.

**FIGURE 1:**
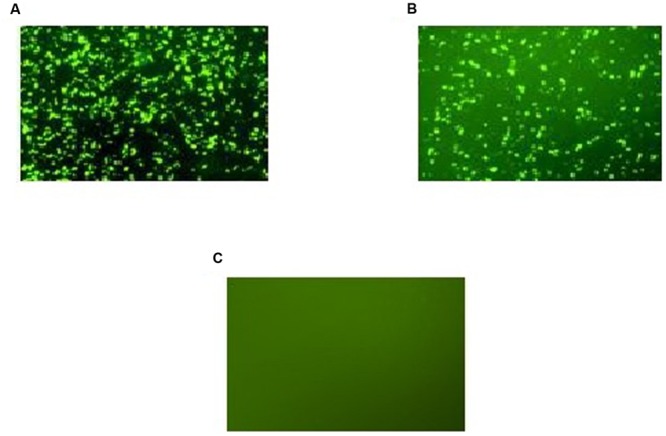
**(A–C)** Expression of recombinant pVAX-1-GFP-ROP1 plasmid in CHO cells at 72 h post-transfection with: **(A)** pVAX1-GFP as positive control, **(B)** pVAX1-GFP-ROP1, and **(C)** PBS as negative control. Protein expression was observed under fluorescence microscopy.

**FIGURE 2:**
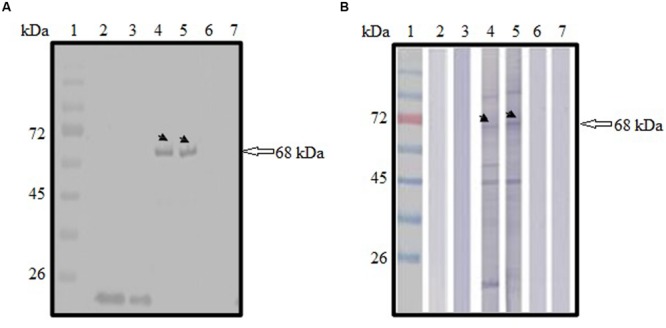
**(A,B)** Immunoblot analysis on the identity and antigenicity of recombinant ROP1 (rROP1) protein expressed in CHO cells. **(A)** Western blot probed with monoclonal anti-GFP antibody. Lanes 2–3 contained cell lysates of pVAX1-GFP-transfected CHO cells. Lanes 4–5 contained cell lysates of pVAX1-GFP-ROP1-transfected CHO cells. Lanes 6–7 contained cell lysates of mock transfection. **(B)** Western blot probed with toxoplasmosis-positive patient serum. Lanes 2–3 contained cell lysates of pVAX1-GFP-transfected CHO cells. Lanes 4–5 contained cell lysates of pVAX1-GFP-ROP1-transfected CHO cells. Lanes 6–7 contained cell lysates of mock transfection. Bound antibodies were detected using alkaline phosphatase-conjugated goat anti-mouse IgG **(A)** and anti-human IgG **(B)**, respectively. Lane 1 **(A,B)** contained protein molecular weight markers. The 68 kDa rROP1 protein was detected (arrow).

Expression of GFP-tagged ROP1 protein was observed fluorescing in green under inverted fluorescence microscopy compared to the negative control (**Figure 1**). Expression of GFP protein in the pVAX1-GFP-transfected CHO cells served as positive control. GFP acts as a biosensor that helps to identify protein expression in mammalian cells which will exhibits green fluorescence when exposed to light in the ultraviolet region. The identity of the expressed rROP1 protein was further confirmed by western blotting against monoclonal anti-GFP antibody and toxoplasmosis-positive human serum. A protein band at the size of approximately 68 kDa was detected corresponding to the predicted size of GFP-tagged rROP1 protein (**Figure 2**). The protein size is about 27 kDa greater than that of the native ROP1 due to the fusion of the 24 kDa GFP protein to the rROP1 protein as well as the post-translational modification factor. Similarly, ROP1 was successfully expressed in mammalian expression vector pCMV-Tag2B with the native size of 43 kDa and was found to be immunogenic *in vivo* and *in vitro* (Quan et al., 2012).

### Prokaryotic Expression and Purification of rROP1

**FIGURE 3:**
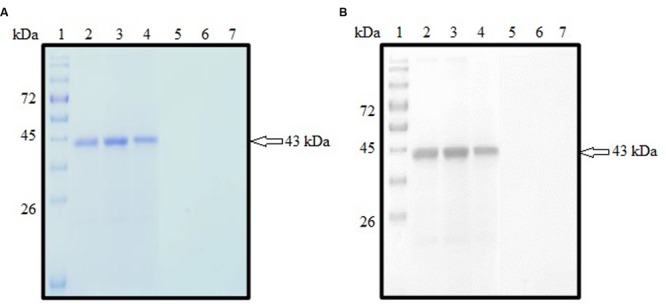
**(A,B)** SDS-PAGE analysis on purified rROP1 protein. **(A)** Coomassie blue stained. Lanes 2–4 contained purified fractions of purified rROP1 protein. Lanes 5–7 contained purified fractions of negative control. **(B)** Western blot probed with monoclonal anti-His antibody. Lanes 2–4 contained purified fractions of purified rROP1 protein. Lanes 5–7 contained purified fractions of negative control. Lane 1 **(A,B)** contained protein molecular weight markers. The 43 kDa purified rROP1 was detected (arrow).

SDS-PAGE analysis showed that rROP1 was successfully expressed and purified with target size of approximately 43 kDa as depicted in **Figure 3A** with the purified vector protein as negative control. Meanwhile, monoclonal anti-His antibody detected a band with the similar size as shown in **Figure 3B**, thereby confirming the identity of rROP1 fused with the His-tag.

The pCold I vector used to clone the target *ROP1* gene contained *cspA* gene encoding for cold-shock protein which is responsible for protein expression at low temperature thereby preventing production of insoluble protein. Soluble rROP1 of approximate protein size of 43 kDa containing the polyhistidine tag (Hengen, 1995) was expressed with optimal IPTG concentration of 1 mM and was purified based on affinity chromatography under native condition. The purification resins involved Ni^2+^ ions that bind to the histidine tag within rROP1 followed by elution of the bound rROP1 by imidazole salts.

### Humoral Immune Response Elicited by the Immunized Mice

#### pVAX1-ROP1

**FIGURE 4:**
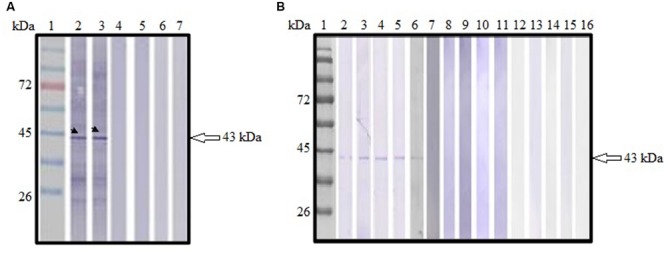
**(A,B)** Immunoblot analysis of antibody production in the vaccinated mice. **(A)** Detection of specific anti-TLA antibody in DNA vaccinated mice sera. Lanes 2–3 contained pVAX1-ROP1-immunized mice sera. Lanes 4–5 contained pVAX1-injected mice sera. Lanes 6–7 contained PBS-injected mice sera. Representative results from two of the 12 mice tested are presented. **(B)** Detection of specific anti-ROP1 antibody in protein vaccinated mice sera. Lanes 2–6 contained rROP1-immunized mice sera. Lanes 7–11 contained vector protein-injected mice sera. Lanes 12–16 contained PBS-injected mice sera. Lane 1 **(A,B)** contained protein molecular weight markers. The 43 kDa ROP1 antigen was detected (arrow).

Sera collected from pVAX1-ROP1 vaccinated mice were shown to react with TLA, detecting a protein size of approximately 43 kDa (**Figure 4A**) which corresponded to the estimated molecular weight of native ROP1. Meanwhile, no specific anti-*Toxoplasma* IgG antibody was detected in sera from both negative control groups (pVAX1 or PBS).

#### rROP1

Sera collected from rROP1 vaccinated mice were shown to react with purified rROP1, detecting a protein size of approximately 43 kDa (**Figure 4B**) which corresponded to the estimated molecular weight of native ROP1. Meanwhile, no specific anti-ROP1 IgG antibody was detected in sera from both negative control groups (pCold I or PBS).

### Lymphoproliferative Response of the Immunized Mice

#### pVAX1-ROP1

**FIGURE 5:**
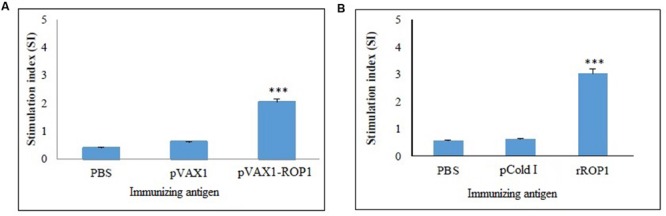
**(A,B)** Cell proliferation assay. *In vitro* splenocytes proliferation assay of mice immunized with **(A)** pVAX1-ROP1 and **(B)** rROP1. The proliferation results (stimulation index) are expressed as mean ± SD (*n* = 3). Statistical difference are represented by ^∗∗∗^*p* < 0.001, in comparison with the respective negative controls.

Single cell suspension of the splenocytes was prepared 2 weeks after final immunization in order to evaluate the proliferative immune response induced. Stimulated spleen cells of the vaccinated mice showed a significant proliferative response to TLA as compared to the non-vaccinated mice (*p* < 0.001) with SI value of 2.04 ± 0.12 (pVAX1-ROP1), 0.6 ± 0.09 (pVAX1), and 0.4 ± 0.04 (PBS; **Table 1** and **Figure 5A**).

**Table 1:** Characterization of cellular-mediated immunity in DNA-vaccinated mice.

Group (*n* = 3)	Proliferation (SI)	Cytokine level (pg/ml)	
l		IFN-γ	IL-4
lpVAX1-ROP1	2.04 ± 0.12***	712 ± 28.1***	94 ± 14.5**
lpVAX1	0.6 ± 0.09	48 ± 10.8	50 ± 13.6
lPBS	0.4 ± 0.04	45 ± 6.6	47 ± 6.1

*SI stands for stimulation index.*

*Data are expressed as mean ± SD (*n* = 3). Statistical difference are represented by ^∗∗∗^*p* < 0.001, in comparison with the control groups (PBS or pVAX1). ^∗∗^ represents *p* < 0.01.*

#### rROP1

Results obtained indicated that the SI value for stimulated splenocytes isolated from mice immunized with rROP1 (3.04 ± 0.21) was significantly higher than that of the mice immunized with vector protein (0.62 ± 0.16) and PBS (0.57 ± 0.04; *p* < 0.001) as shown in **Table 2** and **Figure 5B**.

**Table 2:** Characterization of cellular-mediated immunity in protein-vaccinated mice.

Group (*n* = 3)	Proliferation (SI)	Cytokine level (pg/ml)	
l		IFN-γ	IL-4
lrROP1	3.04 ± 0.21***	1457 ± 31.19***	186 ± 14.17**
lpCold 1	0.62 ± 0.16	102 ± 13.50	86 ± 9.29
lPBS	0.57 ± 0.04	96 ± 10.21	75 ± 6.11

*SI stands for stimulation index.*

*Data are expressed as mean ± SD (*n* = 3). Statistical difference are represented by ^∗∗∗^*p* < 0.001, in comparison with the control groups (PBS or pCold 1). ^∗∗^ represents *p* < 0.01.*

### Cytokines Levels Produced by the Immunized Mice

#### pVAX1-ROP1

**FIGURE 6:**
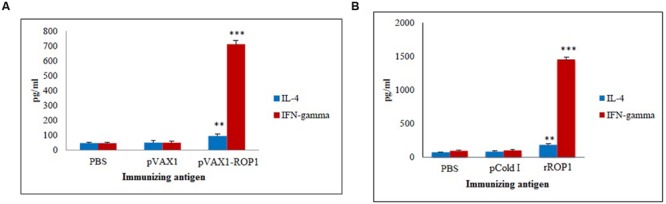
**(A,B)** Cytokine assay. Cytokine production from the stimulated splenocyte culture supernatants of mice immunized with **(A)** pVAX1-ROP1 and **(B)** rROP1. Data are expressed as mean ± SD (*n* = 3). Statistical difference are represented by ^∗∗^*p* < 0.01 or ^∗∗∗^*p* < 0.001, in comparison with the respective negative controls.

Stimulated splenocytes culture supernatants of the immunized mice were harvested 72 h post-induction with TLA to evaluate IFN-γ and IL-4 production level through ELISA. Significantly higher level of IFN-γ (712 ± 28.1 pg/ml) was secreted by the pVAX1-ROP1 immunized mice splenocytes compared to that of pVAX1 (48 ± 10.8 pg/ml) and PBS (45 ± 6.6 pg/ml; *p* < 0.001). On the other hand, higher level of IL-4 was also detected in the splenocytes supernatants from the vaccinated mice (94 ± 14.5 pg/ml) as compared to the negative control groups; 50 ± 13.6 pg/ml and 47 ± 6.1 pg/ml for pVAX1 and PBS, respectively (*p* < 0.01; **Table 1** and **Figure 6A**).

#### rROP1

Stimulated splenocytes culture supernatants of the immunized mice were harvested 72 h post-induction with purified rROP1 to evaluate IFN-γ and IL-4 production level through ELISA. Significantly higher level of IFN-γ (1457 ± 31.19 pg/ml) was secreted by the rROP1 immunized mice splenocytes compared to that of pCold I (102 ± 13.50 pg/ml) and PBS (96 ± 10.21 pg/ml; *p* < 0.001). On the other hand, higher level of IL-4 was detected in the supernatant of splenocytes from the vaccinated mice (186 ± 14.17 pg/ml) as compared to the negative control groups; 86 ± 9.29 pg/ml and 75 ± 6.11 pg/ml for pCold I and PBS, respectively (*p* < 0.01; **Table 2** and **Figure 6B**).

### Protective Immunity against *T. gondii* Challenge

#### pVAX1-ROP1

**FIGURE 7:**
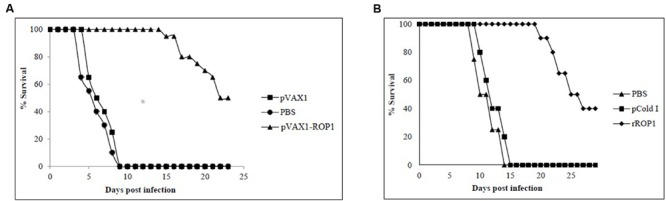
**(A,B)** Study of protective immunity against *T. gondii* infection. Survival rate of mice immunized with **(A)** pVAX1-ROP1 and **(B)** rROP1 after lethal challenge with 1 × 10^3^ tachyzoites compared to the respective negative controls. Each group consisted of 12 mice.

Parasites challenging study revealed that mice immunized with pVAX1 and PBS had a mean survival time of 5 days and all died within the first 9 days. In contrast, mice immunized with pVAX1-ROP1 survived up to 16 days with mean survival time of 23 days (*p* < 0.05). Within the first 9 days, the vaccinated mice had 100% survival rate compared to control mice which all had succumbed to *T. gondii* infection (**Figure 7A**).

#### rROP1

Results indicated that rROP1-immunized mice significantly prolong their survival days (20 days; mean survival of 29 days) in comparison to pCold I- and PBS-injected mice (14 days; mean survival of 7.5 days; *p* < 0.05). Within the first 14 days, the vaccinated mice had 100% survival rate compared to control mice which had 100% mortality (**Figure 7B**).

## Discussion

In our study, mice vaccinated with pVAX1-ROP1 and rROP1 survived longer than the controls when challenged with lethal dose of virulent *T. gondii* RH strain. This protection is likely through humoral and cell-mediated immune responses with secretion of high IFN-γ level but low IL-4 level. An increase in the IFN-γ along with low levels of IL-4 is prerequisite for a Th1-dependent protective immune response (Yan et al., 2012; Parthasarathy et al., 2013). There was a significant correlation between IFN-γ level and degree of protection conferred, suggesting IFN-γ-dependent Th1-predominant immunity is critical for protection against *T. gondii* infection (Ismael et al., 2003; Yuan et al., 2011a). These findings are corresponded to previous reports describing the induction of protective Th1-cell mediated immunity against *T. gondii* infection (Gazzinelli et al., 1991; Denkers and Gazzinelli, 1998; Jordan and Hunter, 2010; Yuan et al., 2011a,b). Survival of the vaccinated mice is mainly dependent on the cellular immunity mediated by both CD4^+^ and CD8^+^ cells through cytokine secretion especially interferon-gamma (IFN-γ). IFN-γ plays a major role in protecting the infected hosts against *T. gondii* infection (Suzuki and Remington, 1990; Gazzinelli et al., 1991). It helps to activate phagocytes such as macrophages to limit the multiplication and spreading of the parasites (Filisetti and Candolfi, 2004). The up-regulation of CD8^+^ T lymphocytes activity in the IFN-γ-treated mice showed that this cytokine is essential for evoking CD8^+^ T cell responses during parasitic infection (Ely et al., 1999). Meanwhile, interleukin-4 (IL-4) that was produced in relatively low level in our study is secreted by CD4^+^ Th2 cells. IL-4 involves in the activation and differentiation of both T and B lymphocytes besides enhancing the MHC-II antigens expression (Filisetti and Candolfi, 2004).

The potential of ROP1 as DNA vaccine candidate has been reported in previous studies (Chen et al., 2001, 2003; Guo et al., 2001; Li et al., 2010; Hiszczynska-Sawicka et al., 2011a; Eslamirad et al., 2012; Quan et al., 2012). Partial ROP1 sequence (765 bp) was cloned into pcDNA vector to construct recombinant DNA for vaccination in mice, in which high IFN-γ level was produced together with increased proliferation of splenocytes (Chen et al., 2001; Guo et al., 2001). It was also reported that DNA vaccine encoding the truncated ROP1 fragment incorporated with SAG1 that was cloned in pEGFP-N3 vector successfully triggered both humoral- and cellular-mediated immune response in the vaccinated mice (Chen et al., 2003). However, challenge study involving the vaccinated mice with *T. gondii* live parasites were not performed in these studies. Meanwhile, Th1-mediated immunity was elicited by recombinant pcDNA-ROP1 (ROP1 partial sequence) DNA plasmid-immunized BALB/c mice with elevated IFN-γ level but low IL-4 production. However, it fails to protect the vaccinated mice against lethal dose (10^5^) of virulent RH strain of *T. gondii* (Eslamirad et al., 2012). In contrast to our study, we had generated pVAX1-ROP1 using full length ROP1 sequence and involved lower dose (10^3^) of the similar parasitic strain. Similarly to our study, full length ROP1 sequence was employed and cloned in different vector (pCMV-Tag2B) before immunizing experimental mice model which induced same type of immunity. Nevertheless, different *T. gondii* strain which is less virulent (ME49) was used to challenge the immunized mice (Quan et al., 2012). In addition, several other rhoptry genes have also been evaluated in DNA vaccination studies such as ROP8, ROP16, and ROP18 delivered via pVAX1 eukaryotic expression vector with slightly higher survival times reported (Yuan et al., 2011a,b; Parthasarathy et al., 2013).

On the other hand, protective efficacy of the subunit vaccines of *Toxoplasma* recombinant antigens produced in bacterial cells was evaluated against *T. gondii* infection in the experimental mice as well which was able to demonstrate certain degree of immunoprotections (Mishima et al., 2001; Martin et al., 2004; Yang et al., 2004; Dziadek et al., 2012; Ching et al., 2016). Various combinations of subunit vaccines using rROP2, rROP4, rGRA4, and rSAG1 have been tested, and it was the rROP2+rROP4+rSAG1 combination conferred the strongest protection against chronic toxoplasmosis in mice (Dziadek et al., 2012). Another study reported generation of mix mode Th1/Th2-immune responses primarily Th1 in the rGRA2 and rGRA5-vaccinated mice with prolonged survival rates against acute toxoplasmosis (Ching et al., 2016). Nevertheless, this study is the first report depicting the immunogenicity and protectivity of rROP1 subunit vaccine against acute toxoplasmosis in the mouse model.

## Conclusion

Our study found strong and specific immunity induced by the ROP1 based vaccine (DNA plasmid and protein), and partial protection was obtained in vaccinated BALB/c mice. Therefore, ROP1 should be considered as a potential vaccine candidate for toxoplasmosis. Complete protection may be achieved by combining ROP1 with other immunogenic rhoptry antigens.

## Author Contributions

YL conceived and designed the study. PS performed all the experiments and analyzed the data. PS and XC drafted the manuscript. YL, MF, and RK critically revised the manuscript. All authors read and approved the final manuscript.

## Conflict of Interest Statement

The authors declare that the research was conducted in the absence of any commercial or financial relationships that could be construed as a potential conflict of interest.

## References

[B1] Abbas A. K. Murphy K. M. Sher A. (1996). Functional diversity of helper T lymphocytes. *Nature* 383 787–793. 10.1038/383787a0 8893001

[B2] Bourguin I. Chardes T. Bout D. (1993). Oral immunization with *Toxoplasma gondii* antigens in association with cholera toxin induces enhanced protective and cell-mediated immunity in C57BL/6 mice. *Infect. Immun.* 61 2082–2088. 8478097 10.1128/iai.61.5.2082-2088.1993 PMC280806

[B3] Buxton D. (1998). Protozoan infections (*Toxoplasma gondii, Neospora caninum* and *Sarcocystis* spp.) in sheep and goats: recent advances. *Vet. Res.* 29 289–310. 9689743

[B4] Buxton D. Thomson K. Maley S. Wright S. Bos H. J. (1991). Vaccination of sheep with a live incomplete strain (S48) of *Toxoplasma gondii* and their immunity to challenge when pregnant. *Vet. Rec.* 129 89–93. 10.1136/vr.129.5.89 1926725

[B5] Carey K. L. Jongco A. M. Kim K. Ward G. E. (2004). The *Toxoplasma gondii* rhoptry protein ROP4 is secreted into the parasitophorous vacuole and becomes phosphorylated in infected cells. *Eukaryot. Cell* 3 1320–1330. 10.1128/EC.3.5.1320-1330.2004 15470260 PMC522600

[B6] Chen G. Guo H. Lu F. Zheng H. (2001). Construction of a recombinant plasmid harbouring the rhoptry protein 1 gene of *Toxoplasma gondii* and preliminary observations on DNA immunity. *Chin. Med. J. (Engl.)* 114 837–840. 11780362

[B7] Chen H. Chen G. Zheng H. Guo H. (2003). Induction of immune responses in mice by vaccination with Liposome-entrapped DNA complexes encoding *Toxoplasma gondii* SAG1 and ROP1 genes. *Chin. Med. J. (Engl.)* 116 1561–1566. 14570624

[B8] Ching X. T. Fong M. Y. Lau Y. L. (2016). Evaluation of immunoprotection conferred by the subunit vaccines of GRA2 and GRA5 against acute toxoplasmosis in BALB/c mice. *Front. Microbiol.* 7:609 10.3389/fmicb.2016.00609 PMC4847622 27199938

[B9] Denkers E. Y. Gazzinelli R. T. (1998). Regulation and function of T-cell-mediated immunity during *Toxoplasma gondii* infection. *Clin. Microbiol. Rev.* 11 569–588. 9767056 10.1128/cmr.11.4.569 PMC88897

[B10] Di Cristina M. Del Porto P. Buffolano W. Beghetto E. Spadoni A. Guglietta S. (2004). The *Toxoplasma gondii* bradyzoite antigens BAG1 and MAG1 induce early humoral and cell-mediated immune responses upon human infection. *Microbes Infect.* 6 164–171. 10.1016/j.micinf.2003.11.009 14998514

[B11] Dlugonska H. (2008). Toxoplasma rhoptries: unique secretory organelles and source of promising vaccine proteins for immunoprevention of toxoplasmosis. *J. Biomed. Biotechnol.* 2008:632424 10.1155/2008/632424 PMC2486357 18670609

[B12] Dziadek B. Gatkowska J. Grzybowski M. Dziadek J. Dzitko K. Dlugonska H. (2012). *Toxoplasma gondii*: the vaccine potential of three trivalent antigen-cocktails composed of recombinant ROP2, ROP4, GRA4 and SAG1 proteins against chronic toxoplasmosis in BALB/c mice. *Exp. Parasitol.* 131 133–138. 10.1016/j.exppara.2012.02.026 22445587

[B13] Ely K. H. Kasper L. H. Khan I. A. (1999). Augmentation of the CD8+ T cell response by IFN-gamma in IL-12-deficient mice during *Toxoplasma gondii* infection. *J. Immunol.* 162 5449–5454. 10228024

[B14] Eslamirad Z. Dalimi A. Ghaffarifar F. Sharifi Z. Hosseini A. Z. (2012). Induction of protective immunity against toxoplasmosis in mice by immunization with a plasmid encoding Toxoplama gondii ROP1 gene. *Afr. J. Biotechnol.* 11 8735–8741. 10.5897/AJB10.1860

[B15] Filisetti D. Candolfi E. (2004). Immune response to *Toxoplasma gondii*. *Ann. Ist. Super. Sanita* 40 71–80. 15269455

[B16] Gazzinelli R. T. Hakim F. T. Hieny S. Shearer G. M. Sher A. (1991). Synergistic role of CD4+ and CD8+ T lymphocytes in IFN-gamma production and protective immunity induced by an attenuated *Toxoplasma gondii* vaccine. *J. Immunol.* 146 286–292. 1670604

[B17] Golkar M. Shokrgozar M. A. Rafati S. Musset K. Assmar M. Sadaie R. (2007). Evaluation of protective effect of recombinant dense granule antigens GRA2 and GRA6 formulated in monophosphoryl lipid A (MPL) adjuvant against Toxoplasma chronic infection in mice. *Vaccine* 25 4301–4311. 10.1016/j.vaccine.2007.02.057 17418457

[B18] Guo H. Chen G. Lu F. Chen H. Zheng H. (2001). Immunity induced by DNA vaccine of plasmid encoding the rhoptry protein 1 gene combined with the genetic adjuvant of pcIFN-gamma against *Toxoplasma gondii* in mice. *Chin. Med. J. (Engl.)* 114 317–320. 11780322

[B19] Gurunathan S. Klinman D. M. Seder R. A. (2000). DNA vaccines: immunology, application, and optimization*. *Annu. Rev. Immunol.* 18 927–974. 10.1146/annurev.immunol.18.1.927 10837079

[B20] Hengen P. (1995). Purification of his-tag fusion proteins from *Escherichia coli*. *Trends Biochem. Sci.* 20 285–286. 10.1016/S0968-0004(00)89045-3 7667882

[B21] Hiszczynska-Sawicka E. Li H. Xu J. B. Holec-Gasior L. Kur J. Sedcole R. (2011a). Modulation of immune response to *Toxoplasma gondii* in sheep by immunization with a DNA vaccine encoding ROP1 antigen as a fusion protein with ovine CD154. *Vet. Parasitol.* 183 72–78. 10.1016/j.vetpar.2011.06.010 21742437

[B22] Hiszczynska-Sawicka E. Oledzka G. Holec-Gasior L. Li H. Xu J. B. Sedcole R. (2011b). Evaluation of immune responses in sheep induced by DNA immunization with genes encoding GRA1, GRA4, GRA6 and GRA7 antigens of *Toxoplasma gondii*. *Vet. Parasitol.* 177 281–289. 10.1016/j.vetpar.2010.11.047 21251760

[B23] Ismael A. B. Sekkai D. Collin C. Bout D. Mevelec M. N. (2003). The MIC3 gene of *Toxoplasma gondii* is a novel potent vaccine candidate against toxoplasmosis. *Infect. Immun.* 71 6222–6228. 10.1128/IAI.71.11.6222-6228.2003 14573640 PMC219545

[B24] Janeway C. A. Jr. Travers P. Walport M. Shlomchik M. J. (2001). “The destruction of antibody-coated pathogens via Fc receptors,” in *Immunobiology: The Immune System in Health and Disease* 5th Edn Austin P. Lawrence E. (New York, NY: Garland Science).

[B25] Jongert E. Melkebeek V. De Craeye S. Dewit J. Verhelst D. Cox E. (2008). An enhanced GRA1-GRA7 cocktail DNA vaccine primes anti-Toxoplasma immune responses in pigs. *Vaccine* 26 1025–1031. 10.1016/j.vaccine.2007.11.058 18221825

[B26] Jongert E. Roberts C. W. Gargano N. Forster-Waldl E. Petersen E. (2009). Vaccines against *Toxoplasma gondii*: challenges and opportunities. *Mem. Inst. Oswaldo Cruz* 104 252–266. 10.1590/S0074-02762009000200019 19430651

[B27] Jordan K. A. Hunter C. A. (2010). Regulation of CD8+ T cell responses to infection with parasitic protozoa. *Exp. Parasitol.* 126 318–325. 10.1016/j.exppara.2010.05.008 20493842 PMC2934887

[B28] Kaplan E. L. Meier P. (1958). Nonparametric estimation from incomplete observations. *J. Am. Stat. Associ.n.* 53 457–481. 10.1080/01621459.1958.10501452

[B29] Khan I. A. Ely K. H. Kasper L. H. (1991). A purified parasite antigen (p30) mediates CD8+ T cell immunity against fatal *Toxoplasma gondii* infection in mice. *J. Immunol.* 147 3501–3506. 1940350

[B30] Kim K. Weiss L. M. (2004). *Toxoplasma gondii*: the model apicomplexan. *Int. J. Parasitol.* 34 423–432. 10.1016/j.ijpara.2003.12.009 15003501 PMC3086386

[B31] Lau Y. L. Thiruvengadam G. Lee W. W. Fong M. Y. (2011). Immunogenic characterization of the chimeric surface antigen 1 and 2 (SAG1/2) of *Toxoplasma gondii* expressed in the yeast *Pichia pastoris*. *Parasitol. Res.* 109 871–878. 10.1007/s00436-011-2315-6 21455621

[B32] Leyva R. Herion P. Saavedra R. (2001). Genetic immunization with plasmid DNA coding for the ROP2 protein of *Toxoplasma gondii*. *Parasitol. Res.* 87 70–79. 10.1007/s004360000296 11199854

[B33] Li B. Oledzka G. Mcfarlane R. G. Spellerberg M. B. Smith S. M. Gelder F. B. (2010). Immunological response of sheep to injections of plasmids encoding *Toxoplasma gondii* SAG1 and ROP1 genes. *Parasite Immunol.* 32 671–683. 10.1111/j.1365-3024.2010.01228.x 20691019

[B34] Liu Q. Singla L. D. Zhou H. (2012). Vaccines against *Toxoplasma gondii*: status, challenges and future directions. *Hum. Vaccin. Immunother.* 8 1305–1308. 10.4161/hv.21006 22906945 PMC3579912

[B35] Lourenco E. V. Bernardes E. S. Silva N. M. Mineo J. R. Panunto-Castelo A. Roque-Barreira M. C. (2006). Immunization with MIC1 and MIC4 induces protective immunity against *Toxoplasma gondii*. *Microbes Infect.* 8 1244–1251. 10.1016/j.micinf.2005.11.013 16616574

[B36] Martin V. Supanitsky A. Echeverria P. C. Litwin S. Tanos T. De Roodt A. R. (2004). Recombinant GRA4 or ROP2 protein combined with alum or the gra4 gene provides partial protection in chronic murine models of toxoplasmosis. *Clin. Diagn. Lab. Immunol.* 11 704–710. 15242945 10.1128/CDLI.11.4.704-710.2004 PMC440599

[B37] Mevelec M. N. Bout D. Desolme B. Marchand H. Magne R. Bruneel O. (2005). Evaluation of protective effect of DNA vaccination with genes encoding antigens GRA4 and SAG1 associated with GM-CSF plasmid, against acute, chronical and congenital toxoplasmosis in mice. *Vaccine* 23 4489–4499. 10.1016/j.vaccine.2005.04.025 15935521

[B38] Mishima M. Xuan X. Shioda A. Omata Y. Fujisaki K. Nagasawa H. (2001). Modified protection against *Toxoplasma gondii* lethal infection and brain cyst formation by vaccination with SAG2 and SRS1. *J. Vet. Med. Sci.* 63 433–438. 10.1292/jvms.63.433 11346179

[B39] Parthasarathy S. Fong M. Y. Ramaswamy K. Lau Y. L. (2013). Protective immune response in BALB/c mice induced by DNA vaccine of the ROP8 gene of *Toxoplasma gondii*. *Am. J. Trop. Med. Hyg.* 88 883–887. 10.4269/ajtmh.12-0727 23509124 PMC3752752

[B40] Peng G. H. Yuan Z. G. Zhou D. H. He X. H. Liu M. M. Yan C. (2009). *Toxoplasma gondii* microneme protein 6 (MIC6) is a potential vaccine candidate against toxoplasmosis in mice. *Vaccine* 27 6570–6574. 10.1016/j.vaccine.2009.08.043 19720368

[B41] Quan J.-H. Chu J.-Q. Ismail H. A. H. A. Zhou W. Jo E.-K. Cha G.-H. (2012). Induction of protective immune responses by a multiantigenic DNA vaccine encoding GRA7 and ROP1 of *Toxoplasma gondii*. *Clin. Vacci. Immunol.* 19 666–674. 10.1128/CVI.05385-11 PMC3346315 22419676

[B42] Robinson H. L. (1999). DNA vaccines: basic mechanism and immune responses (Review). *Int. J. Mol. Med.* 4 549–555. 10534580 10.3892/ijmm.4.5.549

[B43] Romagnani S. (1994). Lymphokine production by human T cells in disease states. *Annu. Rev. Immunol.* 12 227–257. 10.1146/annurev.iy.12.040194.001303 8011282

[B44] Saeij J. P. Boyle J. P. Coller S. Taylor S. Sibley L. D. Brooke-Powell E. T. (2006). Polymorphic secreted kinases are key virulence factors in toxoplasmosis. *Science* 314 1780–1783. 10.1126/science.1133690 17170306 PMC2646183

[B45] Sun X. M. Zou J. Aa E. S. Yan W. C. Liu X. Y. Suo X. (2011). DNA vaccination with a gene encoding *Toxoplasma gondii* GRA6 induces partial protection against toxoplasmosis in BALB/c mice. *Parasit. Vectors* 4:213 10.1186/1756-3305-4-213 PMC3229464 22070984

[B46] Suzuki Y. Remington J. S. (1990). The effect of anti-IFN-gamma antibody on the protective effect of Lyt-2+ immune T cells against toxoplasmosis in mice. *J. Immunol.* 144 1954–1956. 2106557

[B47] Weeks-Levy C. Tatem J. M. Dimichele S. J. Waterfield W. Georgiu A. F. Mento S. J. (1991). Identification and characterization of a new base substitution in the vaccine strain of Sabin 3 poliovirus. *Virology* 185 934–937. 10.1016/0042-6822(91)90576-W 1660210

[B48] Yan H. K. Yuan Z. G. Song H. Q. Petersen E. Zhou Y. Ren D. (2012). Vaccination with a DNA vaccine coding for perforin-like protein 1 and MIC6 induces significant protective immunity against *Toxoplasma gondii*. *Clin. Vacci. Immunol.* 19 684–689. 10.1128/CVI.05578-11 PMC3346325 22379063

[B49] Yang C. D. Chang G. N. Chao D. (2004). Protective immunity against *Toxoplasma gondii* in mice induced by a chimeric protein rSAG1/2. *Parasitol. Res.* 92 58–64. 10.1007/s00436-003-0992-5 14605877

[B50] Yuan Z. G. Zhang X. X. He X. H. Petersen E. Zhou D. H. He Y. (2011a). Protective immunity induced by *Toxoplasma gondii* rhoptry protein 16 against toxoplasmosis in mice. *Clin. Vacci. Immunol.* 18 119–124. 10.1128/CVI.00312-10 PMC3019779 21106780

[B51] Yuan Z. G. Zhang X. X. Lin R. Q. Petersen E. He S. Yu M. (2011b). Protective effect against toxoplasmosis in mice induced by DNA immunization with gene encoding *Toxoplasma gondii* ROP18. *Vaccine* 29 6614–6619. 10.1016/j.vaccine.2011.06.110 21762755

